# Changes in the use of diabetes drugs among community-dwelling people with Alzheimer’s disease

**DOI:** 10.1186/s12877-021-02694-w

**Published:** 2021-12-15

**Authors:** Carlos López-Rubio, Marjaana Koponen, Pasi Lampela, Heidi Taipale, Antti Tanskanen, J. Simon Bell, Anna-Maija Tolppanen, Sirpa Hartikainen

**Affiliations:** 1 School of Medicine, University of Eastern Finland, Kuopio, Finland; 2 Kuopio Research Centre of Geriatric Care, University of Eastern Finland, Kuopio, Finland; 3 School of Pharmacy, University of Eastern Finland, PO Box 1627, 70210 Kuopio, Finland; 4 Centre for Medicine Use and Safety, Faculty of Pharmacy and Pharmaceutical Sciences, Monash University, Parkville, Victoria Australia; 5 Department of Clinical Neuroscience, Karolinska Institutet, Stockholm, Sweden; 6 Department of Forensic Psychiatry, Niuvanniemi Hospital, University of Eastern Finland, Kuopio, Finland

## Abstract

**Background:**

Type 2 diabetes is common in persons with Alzheimer’s disease (AD). Management of diabetes in persons with AD is challenging due to changing goals of care and susceptibility to adverse drug events including hypoglycemia. The aim of this study was to investigate the prevalence of diabetes drug use from 5 years before to 5 years after the time of AD diagnosis among persons with and without AD.

**Methods:**

This was a nationwide register-based study of persons with and without AD and diabetes in Finland. We analyzed data from the Medication Use and Alzheimer’s disease (MEDALZ) study that included 70,718 community-dwelling people diagnosed with AD from 2005 to 2011. The study population included 8418 persons with AD and 6666 matched persons without AD who were diagnosed with diabetes 5 years before AD diagnosis (index date). We defined the prevalence of diabetes drug use in three-month evaluation periods from 5 years before until 5 years after the index date.

**Results:**

Nearly all people with diabetes (94% in both cohorts) used one or more diabetes drugs on the index date. The most prevalent drug metformin was used by 60.9% of people with AD and 59.1% of people without AD. The next most prevalent drugs were sulfonylureas and insulin. The prevalence of diabetes drug use was similar in people with and without AD but began to decline 1 year after AD diagnosis in the AD cohort compared to non-AD cohort.

**Conclusions:**

The decline in diabetes drug use after AD diagnosis may be attributed to clinicians and patients seeking to avoid serious adverse drug events including hypoglycemia. In addition, the findings may reflect personalized glycemic control and unintentional weight loss in persons with AD reducing the need for diabetes drugs.

**Supplementary Information:**

The online version contains supplementary material available at 10.1186/s12877-021-02694-w.

## Introduction

Alzheimer’s disease (AD) is a neurodegenerative disease and most common form of dementia that predominately affects older people [1]. The global prevalence of type 2 diabetes has increased in recent decades and 422 million people now live with type 1 or type 2 diabetes [2]. Nearly 30% of persons with diabetes were aged 65 years and older in 2019 [3]. About one quarter (26.8%) of Americans aged 65 years and older have type 2 diabetes [4]. Both AD and diabetes share several risk factors [5] and their coexistence is common [6]. The benefits and risks of intensive glycemic control in persons with AD may be different to that of the general population.

Management of diabetes and self-care is challenging for persons with AD and their caregivers. Persons with AD may forget to eat meals, experience challenges taking medication, and have difficulties in recognizing symptoms of hypoglycemia [7]. Clinical practice guidelines for managing diabetes in persons with cognitive impairment emphasize the importance of an individualized approach focused on maintaining quality of life through preventing hypoglycemia and reducing hyperglycemia [8]. This includes providing care across a continuum with less stringent HbA1c lowering for people with advanced dementia or who are frail. Deintensification of diabetes treatment regimens may include discontinuing drugs most likely to cause hypoglycemia (e.g. sulfonylureas, insulin) or switching to agents associated with a lower risk of hypoglycemia [9].

However, there is a lack of data on the use of diabetes drugs after AD diagnosis. One prospective study of persons with type 2 diabetes and dementia reported the total number of diabetes drugs but did not analyze drug groups individually [10]. To our knowledge, no previous studies have investigated changes in the use of diabetes drugs among people with dementia. The aim of this study was to investigate the temporal changes in prevalence of diabetes drugs in relation to AD diagnosis, and compare the changes in prevalence to a comparison cohort of persons without AD.

## Methods

### Study cohort

We analyzed data from the register-based MEDALZ (Medication Use and Alzheimer’s disease) cohort that included 70,718 community-dwelling residents of Finland who received a verified diagnosis of AD from 2005 to 2011 [11]. Each person with AD was matched to a comparison person without AD according to age-, sex- and region of residence (*n* = 70,718). Persons with AD were identified from the Special Reimbursement Register maintained by the Social Insurance Institution of Finland (SII). AD was diagnosed according to NINCDS-ADRDA [12] and DSM-IV criteria. For a diagnosis of AD to be verified by the SII a person needed to fulfil all the following criteria: (i) symptoms consistent with AD; (ii) a decrease in social capacity over a period of at least 3 months; (iii) received a computed tomography/magnetic resonance imaging scan; (iv) had possible alternative diagnoses excluded; and (v) received confirmation of the diagnosis by a registered geriatrician or neurologist. Additional data were obtained from the Finnish nationwide registers including information on chronic comorbidities from the Special Reimbursement Register (1972–2015), hospital admissions with diagnosis from the Hospital Discharge Register (1972–2015) and reimbursed prescription drug dispensing from the Prescription Register (1995–2015).

### Study design

The study sample included all persons with and without AD with diabetes 5 years before the date of AD diagnosis (index date). Persons were considered to have diabetes if they were eligible for special reimbursement of diabetes drugs by the SII and/or if they had been dispensed reimbursed diabetes drugs according to Anatomical Therapeutic Chemical (ATC) classification [13]. The date for diabetes diagnosis was defined either as the date of entitlement for reimbursement, or the date of first purchase of diabetes drug, whichever occurred first.

Use of diabetes drugs were modelled from drug dispensings recorded in the Prescription Register with validated ‘Prescriptions to drug use periods’ (PRE2DUP) model [14]. This model considers regularity of drug dispensing, stockpiling of drugs and possible hospital/institutional care when drugs are provided by the caring unit. Previous research has demonstrated good agreement between PRE2DUP modelled drug use and self-reported use of insulin and oral diabetes drugs [15]. Diabetes drugs were defined as insulins, metformin, sulfonylureas and other diabetes drugs including sulfonamides, alpha glucosidase inhibitors, thiazolidinediones, dipeptidyl peptidase 4 inhibitors (DPP-4), glucagon-like peptide-1 analogues (GPL-1), sodium-glucose co-transporter 2 inhibitors (SGLT2), and repaglinide, nateglinide, pramlintide, benfluorex and mitiglinide (Supplementary Table 1). The prevalence of diabetes drug use was defined in three-month evaluation periods from 5 years before until 5 years after the index date. Drugs used in public nursing homes and hospitals are not recorded in the Prescription Register. Therefore, only persons who were alive and not hospitalized nor a nursing home resident for more than 30 days of a specific evaluation period were included. Follow-up ended on the date of death, 5 years after index date, or at the end of data linkage (31 Dec 2015), whichever occurred first. In addition, persons in the non-AD cohort were censored from the study at their AD diagnosis date if they received AD diagnosis during the follow-up.

Under Finnish legislation, ethics committee approval was not required because no participants were contacted and data were de-identified by the data custodians prior to analyses by the researchers.

### Statistical analysis

Characteristics of persons with AD and matched comparison persons without AD were presented using frequencies and percentages or means and standard deviations (SD). Categorical variables were compared by the Chi-square test and continuous variables by the T-test. The prevalence of diabetes drug use in each observation period were presented with 95% confidence intervals. All analyses were conducted using SPSS software (IBM SPSS Statistics Version 25) for Windows.

## Results

There were 8418 persons with AD and 6666 persons without AD who had diabetes 5 years before the index date included in the study. The mean age was similar in both groups (79.9 years and 80.7 years for persons with and without AD, respectively) and the majority of persons were women in both cohorts (59.2% and 63.0 in AD and non-AD cohorts) (Supplementary Table 2).

**Fig. 1:**
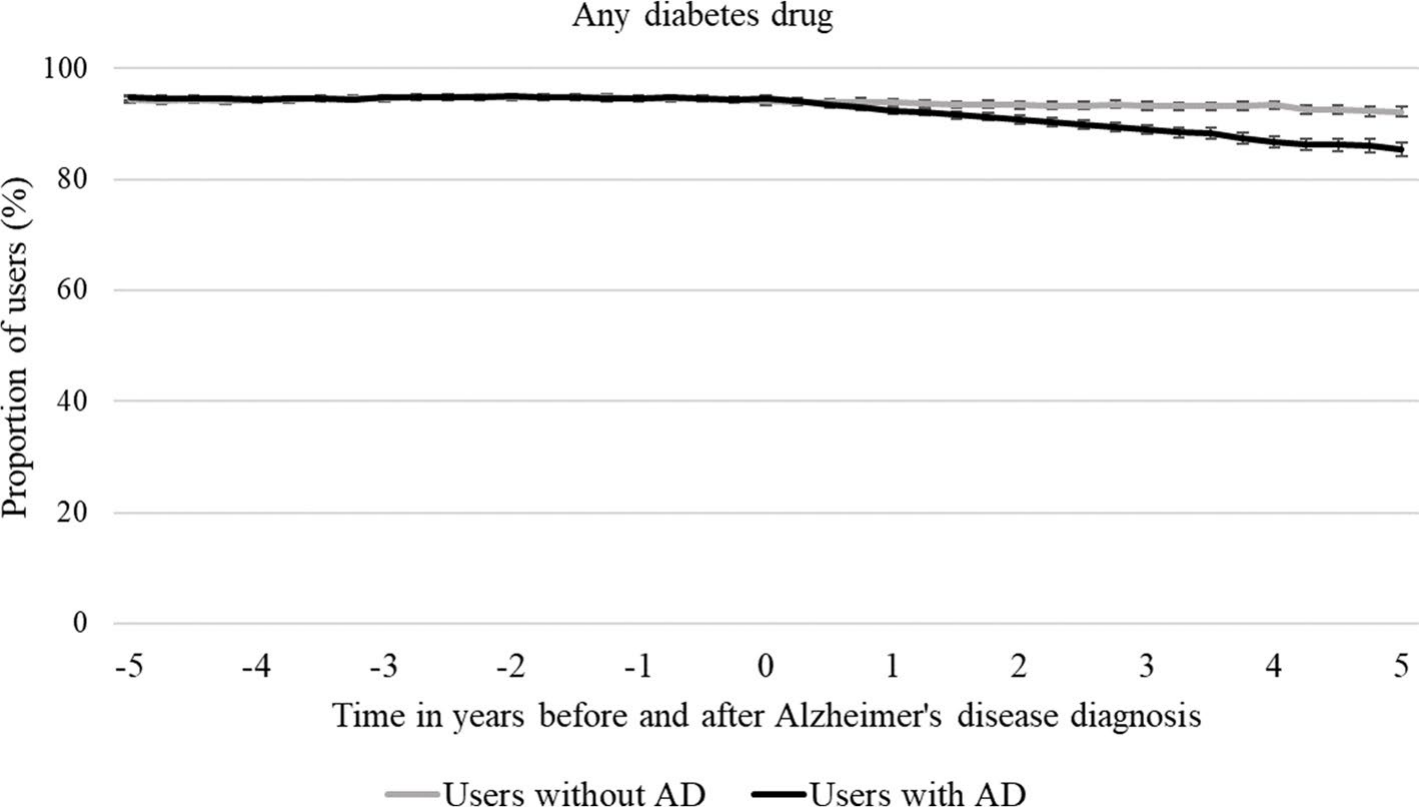
Prevalence of any diabetes drug use in persons with and without Alzheimer’s disease (AD) with 95% confidence intervals

Nearly all persons with diabetes (94.6% of the AD-cohort and 94.0% of non-AD cohort) used at least one diabetes drug on the index date (Supplementary Table 2). Metformin was the most prevalent diabetes drug (60.9 and 59.1% in AD- and non-AD cohorts, respectively) followed by sulfonylureas and insulin. There were no differences in the prevalence of diabetes drug use between persons with and without AD prior to the index date (Fig. 1). The prevalence of diabetes drug use started to decline continuously 1 year after AD diagnosis until the end of follow-up in persons with AD. In contrast, the prevalence of diabetes drug use remained constant in the comparison persons without AD. At the end of the follow-up the prevalence of diabetes drug use was higher in people without AD 91.17% (95% Cl 91.26–93.10 compared to those with AD 85.33% (95% Cl 84.33–86.62).

**Fig. 2:**
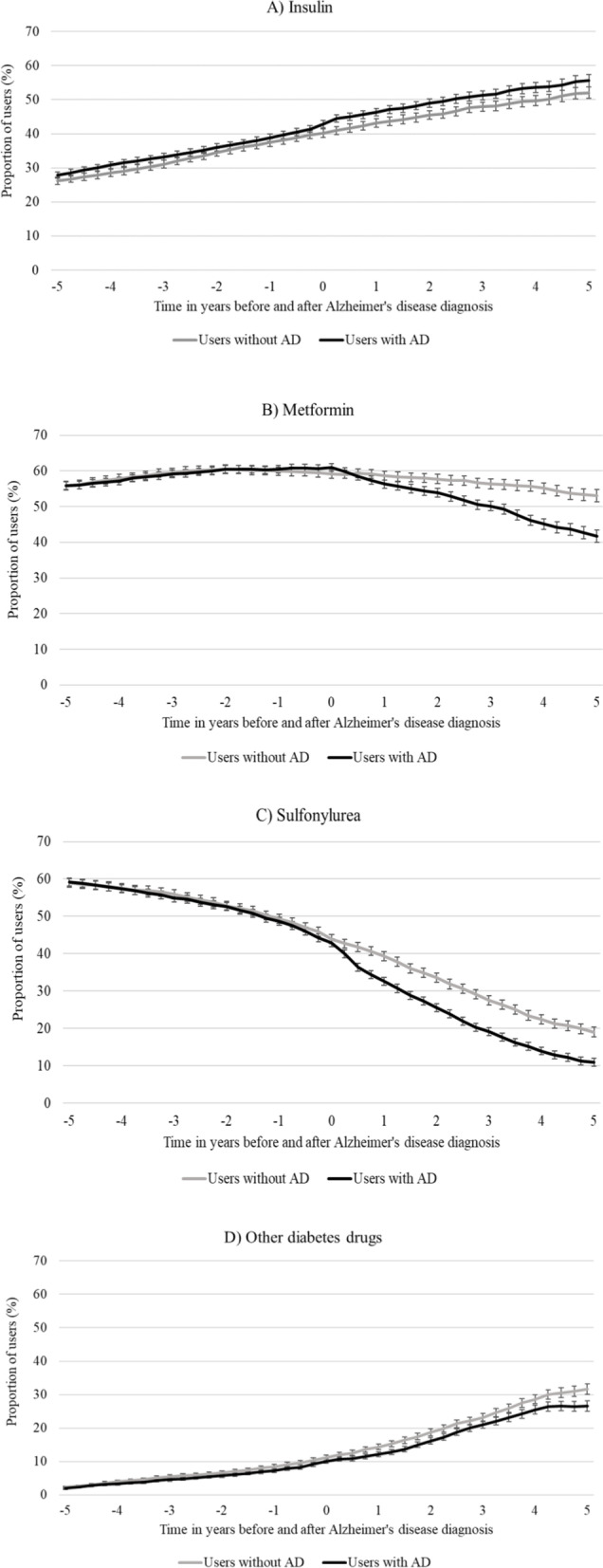
Prevalence of use of a) insulin, b) metformin, c) sulfonylureas and d) other diabetes drugs in the cohort of persons without and with Alzheimer’s disease (AD) with 95% intervals

The proportion of persons using insulin increased throughout the 10-year follow-up in both cohorts (from 26.2 to 51.8% and from 27.9 to 55.6% in cohorts without and with AD, respectively) (Fig. 2A). The prevalence of metformin use decreased in both the AD and non-AD cohorts, although this decrease was more pronounced in the AD cohort (Fig. 2B). The use of sulfonylureas decreased in both cohorts throughout the 10-year follow-up, but this decrease was also more pronounced in the AD cohort (Fig. 2C). Use of other diabetes drugs (including sulfonamides, alpha glucosidase inhibitors, thiazolidinediones and DPP-4 inhibitors, GLP-1 analogues, SGLT2 inhibitors) was rare (1.9% in persons with AD, 2.2% in persons without AD) at the beginning of the follow-up but increased over 10-fold until the end of the follow-up (26.6% in AD and 31.5% in non-AD cohort, respectively) (Fig. 2D). The increase was primarily attributable to increased use of DPP-4 inhibitors, as the use of other diabetes drugs was minimal (data not shown). After the index date the increase in use was lower among people with AD.

**Fig. 3:**
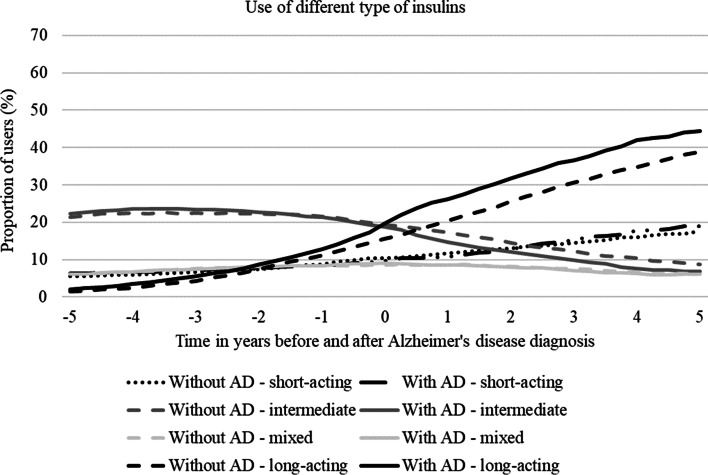
Prevalence of use by different insulin types in the cohort without and with Alzheimer’s disease (AD)

Use of short- and particularly long-acting insulins increased throughout the 10-year period in both cohorts (Fig. 3). The increase of long-acting insulins was more pronounced in persons with AD at the year of AD diagnosis and remained higher than in persons without AD throughout the follow-up. Use of intermediate-acting insulin decreased over the follow-up and this decrease was more evident after index date among persons with AD. Use of mixed insulins was relatively stable in both cohorts over the follow-up.

## Discussion

To our knowledge, this is the first study to report changes in the use of diabetes drugs in persons with and without AD. Diabetes drug use decreased one year after diagnosis in persons with AD compared to those without AD. This finding is in accordance with a previous United States study on changes on drug utilization among people with diabetes following AD diagnosis [10]. The decrease in diabetes drug use may be partly attributable to unintentional weight loss and a corresponding reduction in the need for diabetes drugs after AD diagnosis [16, 17]. The decrease might also reflect recognition that tight glycemic control in vulnerable older people can lead to hypoglycemia [18], increased risk of falls and related fractures, micro- and macrovascular complications or even death [19]. In addition, weight loss is related to frailty which has been demonstrated to increase the risk of hypoglycemia in persons with dementia [20] Severe hypoglycemia is more common in people with diabetes and dementia compared to those without dementia [21]. Therefore, optimizing diabetes treatment regimens according to each individual’s glycemic goals is encouraged in persons with cognitive impairment [18]. This approach is consistent with a focus on maintaining quality of life through avoiding hypoglycemia [7]. Our results are in contrast to a recent Australian study that reported clinicians appeared to prescribe more conservatively for people with diabetes who were older and frailer, but not for people with dementia [22].

In our study, metformin was the most frequently used diabetes drug at the index date among people with and without AD. Metformin has a favorable benefit-to-risk ratio although should be used with caution in renal impairment due to the risk of lactic acidosis [23, 24]. However, with appropriate dose adjustment metformin has been shown to be largely safe and effective in people with chronic kidney disease [25]. This is in line with guidelines recommending metformin as the first-line agent for type 2 diabetes [26]. This likely explains the frequent use of metformin in both cohorts. While these findings were consistent with international findings, older age has also been associated with higher odds of initiating non-metformin treatment [27].

Sulfonylureas have been associated with an increased risk of severe and long-lasting hypoglycemia, which may outweigh their benefits among older people [28, 29]. Furthermore, their beneficial effects may decrease after 2 years of use [26]. Introduction of newer and safer diabetes drugs is likely to explain the decreased use of sulfonylureas over time in both cohorts. The shift away from sulfonylureas may also reflect an international transition toward newer diabetes treatments such as SGLT-2 inhibitors, DPP-4 inhibitors and GLP-1 agonists [30].

The use of long-acting insulin increased throughout the follow-up in people with and without AD. These changes were consistent with recommendations for insulin selection in older people with diabetes and cognitive impairment. Basal insulin is generally considered the insulin of choice due to the less frequent administration and reduced risk of adverse events [7, 31].

DPP-4 inhibitors were the most frequently used newer diabetes drugs whereas use of SGLT-2 inhibitors and thiazolidinediones was minimal. DPP-4 inhibitors have a favorable safety profile among older persons although caution is needed in patients with heart failure, and dose reduction is needed in renal impairment [27]. As these newer diabetes drugs became available during the follow-up, their prevalence was low at the beginning of the follow-up.

The main strength of the study was the large national population-based cohort of community-dwelling persons with a clinically verified diagnosis of AD. Data linkage enabled us to study drug use over a 10-year follow-up and avoid recall and selection biases. However, the Finnish Prescription Register did not include information about drug use during periods persons were in hospitals or public nursing homes. Therefore, we censored persons from the observation period if they stayed in hospitals or nursing homes for more than 30 days during each specific 3-month period. Consequently, our findings are only generalizable to community-dwelling persons. In addition, the Special Reimbursement Register lacked information about the type of diabetes. The registers also lacked the information on the severity of AD and clinical information on short- and long-term levels of blood glucose, glycosylated hemoglobin levels and other clinical factors that were likely to have affected the decision to prescribe and discontinue diabetes drugs. Further research is needed on the optimal management of diabetes in persons with dementia and to develop safe and feasible treatment regimens for this vulnerable population. This includes research into different diabetes treatment deintensification strategies in people with dementia.

## Conclusion and implications

The prevalence of diabetes drug use decreases 1 year following diagnosis of AD compared to matched people not diagnosed with AD. The decrease in the overall use of diabetes drugs and increase in long-acting insulins after AD diagnosis likely reflects clinicians’ and patients’ attempts to avoid hypoglycemia in this vulnerable group of older persons.

## Supplementary Information

**Additional file 1.**

**Additional file 2.**

## Data Availability

Restrictions apply to the availability of the data that support the findings of this study, so they are not publicly available. Data are available from the authors upon reasonable request and if appropriate permission from the Finnish Social and Health Data Permit Authority Findata is sought and granted.

## Abbreviations

AD: Alzheimer’s Disease
ATC: Anatomical Therapeutic Chemical
DPP-4: Dipeptidyl peptidase 4 inhibitors,
DSM-IV: Diagnostic and Statistical Manual of Mental Disorders
GLP-1: Glucagon-like peptide-1 analogues
MEDALZ: Medication Use and Alzheimer’s disease
NINCDS-ADRDA: National Institute of Neurological and Communicative Disorders and Stroke and the Alzheimer’s Disease and Related Disorders Association
SD: Standard Deviation
SGLT2: Sodium-glucose co-transporter 2 inhibitors
SII: Social Insurance Institution
