# Supplementary material for: Changes in the use of diabetes drugs among community-dwelling people with Alzheimer’s disease
Source: BMC Geriatr. 2021 Dec 15;21:701. doi: 10.1186/s12877-021-02694-w (PMC8672592; doi:10.1186/s12877-021-02694-w)
Supplement: Supplementary file 1 — Additional file 1. [file 12877_2021_2694_MOESM1_ESM.docx]

Supplement Table 1. Classification of diabetes drugs according to the ATC-code

| **Diabetes Drugs** | **ATC Codes** |
| --- | --- |
| Metformin | A10BA02, A10BD02-03, A10BD05, A10BD07-08, A10BD10-11, A10BD13 |
| Insulin | A10A |
| Sulfonylureas | A10BB, A10BD02, A10BD04, A10BD06 |
| Sulfonamides | A10BC |
| Alpha glucosidase inhibitors | A10BF |
| Thiazolidinediones | A10BG, A10BD03-6, A10BD09 |
| Dipeptidyl peptidase 4 inhibitors (DPP-4) | A10BH, A10BD07-11, A10BD13 |
| Glucagon-like peptide-1 analogues (GPL-1) | A10BJ |
| Sodium-glucose co-transporter 2 inhibitors (SGLT2) | A10BK |
| Repaglinide | A10BX02 |
| Nateglinide | A10BX03 |
| Pramlintide | A10BX05 |
| Benfluorex | A10BX06 |
| Mitiglinide | A10BX08 |
