# Supplementary material for: Changes in the use of diabetes drugs among community-dwelling people with Alzheimer’s disease
Source: BMC Geriatr. 2021 Dec 15;21:701. doi: 10.1186/s12877-021-02694-w (PMC8672592; doi:10.1186/s12877-021-02694-w)
Supplement: Supplementary file 2 — Additional file 2. [file 12877_2021_2694_MOESM2_ESM.docx]

|  | **Persons with AD**  **(n= 8143)** | **Persons without AD (n=6560)** | **p-value** |
| --- | --- | --- | --- |
|  |  |  |  |
| **Characteristics** |  |  |  |
| **Age, years (n, %)** |  |  | <0.001 |
| < 75 | 1650 (20.3) | 1137 (17.3) |  |
| 75-84 | 4801 (59.0) | 3767 (57.4) |  |
| ≥ 85 | 1692 (20.8) | 1656 (25.2) |  |
| **Sex (n,** %**)** |  |  | <0.001 |
| Women | 4824 (59.2) | 4133 (63.0) |  |
| Men | 3319 (40.8) | 2427 (37.0) |  |
| **Prevalence of diabetes drug use (n, %)** | | | |
| Any diabetes drug | 7702 (94.6) | 6167 (94.0) | 0.134 |
| Insulin | 3489 (42.9) | 2636 (40.2) | 0.001 |
| Metformin | 4961 (60.9) | 3880 (59.2) | 0.029 |
| Sulfonylurea | 3489 (42.9) | 2881 (43.9) | 0.193 |
| Other diabetes drugs | 820 (10.1) | 728 (11.1) | 0.044 |
|  | | | |
|  | | | |

Supplementary Table 2. Characteristics of persons with diabetes and comparison between persons with and without Alzheimer’s disease (AD) and use of diabetes drugs at the index
